# The Association of Hyperuricemia and Gout With the Risk of Cardiovascular Diseases: A Cohort and Mendelian Randomization Study in UK Biobank

**DOI:** 10.3389/fmed.2021.817150

**Published:** 2022-03-23

**Authors:** Jianwei Zhu, Yu Zeng, Hanyue Zhang, Yuanyuan Qu, Zhiye Ying, Yajing Sun, Yao Hu, Wenwen Chen, Huazhen Yang, Jing Yang, Huan Song

**Affiliations:** ^1^Department of Orthopedics, Orthopedic Research Institute, West China Hospital, Sichuan University, Chengdu, China; ^2^West China Biomedical Big Data Center, West China Hospital, Sichuan University, Chengdu, China; ^3^Med-X Center for Informatics, Sichuan University, Chengdu, China; ^4^Division of Nephrology, Kidney Research Institute, State Key Laboratory of Biotherapy and Cancer Center, West China Hospital, Sichuan University, Chengdu, China; ^5^Center of Public Health Sciences, Faculty of Medicine, University of Iceland, Reykjavík, Iceland

**Keywords:** hyperuricemia, gout, cardiovascular diseases, cohort study, Mendelian randomization

## Abstract

**Background:**

The association between hyperuricemia/gout with cardiovascular diseases (CVD) have been investigated. However, whether the magnitude of associations differs between hyperuricemia and gout, and the causality of these associations, remains inconclusive.

**Methods:**

Based on UK Biobank, we conducted a cohort analysis including 431,967 participants, who were categorized as gout, hyperuricemia, and normal groups at recruitment, and followed up for CVD until December 2019. The phenotypic association of hyperuricemia/gout with CVD was estimated by Cox regression, adjusting for multiple confounders. Further exploration on the causality of such links was performed using Mendelian Randomization (MR) analysis, where we selected exclusive genetic variants for hyperuricemia and for gout based on summary GWAS data from independent populations.

**Results:**

During mean 10.20 years of follow-up, hyperuricemia patients were associated with increased CVD (HR = 1.33, 95% CI: 1.29–1.36), compared to individuals who were free of hyperuricemia/gout. The risk elevation was even higher for gout patients (HR = 1.54, 95% CI: 1.48–1.62). Furthermore, we found significantly positive association between genetic liability for hyperuricemia and CVD in both one-sample (OR = 1.06, 95% CI: 1.02–1.11) and two-sample (OR = 1.09, 95% CI: 1.03–1.16) MR analysis. However, genetic liability for gout was not associated with CVD (OR = 0.89, 95% CI: 0.79–1.01 in one-sample, and OR = 0.92, 95% CI: 0.82–1.21 in two-sample MR analysis).

**Conclusion:**

Individuals with hyperuricemia/gout were at increased risk of various types of CVD. As the MR analyses suggest a causal effect of hyperuricemia, but not gout, on CVD, these results indicate the possible effects of other gout-associated factors on the development of CVD, in addition to the uric acid pathway.

## Introduction

The association between hyperuricemia or gout and the risk of cardiovascular diseases (CVDs) have been widely investigated in previous epidemiologic studies (1–4). Specifically, hyperuricemia has been associated with the increased risk of any (2) and specific subtypes of CVD, including stroke (1), coronary heart disease (5), incident hypertension (2), atherosclerosis (6), and atrial fibrillation (7). Likewise, with further enhanced magnitude of association (8), gout was noted as an independent risk factor for coronary heart disease (3, 4, 9), peripheral arterial disease (10), heart failure (11), stroke (12), and CVD mortality (8), suggesting a continuum of increase in CVD risk from hyperuricemia to gout (8). However, with methodological shortcomings of previous studies, such as cross-sectional design (3), selection bias due to various indications for prescription of blood test (3, 4), and insufficient control for important confounders such as lifestyle factors (6, 9, 10), as well as the absence of study examining the differential effects of hyperuricemia and gout on CVD using longitudinal data of the same population, the associations between level of serum urate, gout, and CVD need further assessments, with ideally population-based data and vigorous study design.

Moreover, as the supportive data mainly derived from observational studies, with so far limited knowledge on the underlying mechanisms, the causality between hyperuricemia or gout and CVD remains inconclusive. Using Mendelian randomization (MR) analysis, an approach utilizing genetic instrumental variants associated with the exposure phenotype as a proxy to infer causality (13), previous study showed a potential causality between hyperuricemia with hypertension and myocardial infarction (14), whereas a recent MR study focusing on hyperuricemia and ischemic heart diseases failed to provide consistent evidence (15). In addition, no well-powered causality assessment was found for gout and CVD comorbidities to date, which leads to uncertainties on the question whether serum urate lowering therapy is enough for preventing CVD-related consequence among patients with gout.

Therefore, taking advantage of the multi-dimensional prospective cohort data in UK Biobank, which provides available information on serum urate level and enriched phenotypical variables collected at baseline, complete follow-up data from linked national health registers, and individual-level genotyping data, for more than half million participants (16), we conducted a cohort analysis to elucidate the association between hyperuricemia, gout and subsequent CVD. We further aimed to examine the causal relation between hyperuricemia/gout and CVD. With additional attempts on distinguishing genetic variants specifically associated to asymptotic hyperuricemia from those to gout in MR analysis, our study explored to what extent the progressive order of these two traits (i.e., a part of individuals with hyperuricemia can progress to gout) attributed to the observed phenotypical associations.

## Methods

### Data Source

UK Biobank is a cohort study where 502,507 participants, aged 40–69 years, were recruited across the UK between 2006 and 2010 (https://www.ukbiobank.ac.uk/) (16). Baseline information including social-demographic characteristics, lifestyle, and environmental factors was collected for all participants at recruitment. Future health and survival status can be monitored through data linkages with multiple national health registers (e.g., inpatient, primary care, and death registers) (17). Moreover, UK Biobank obtained genotyping data from blood samples of 487,409 participants (18).

### Study Design

#### A Cohort for Assessing the Phenotypic Association Between Hyperuricemia/Gout and Subsequent CVD

**Figure 1:**
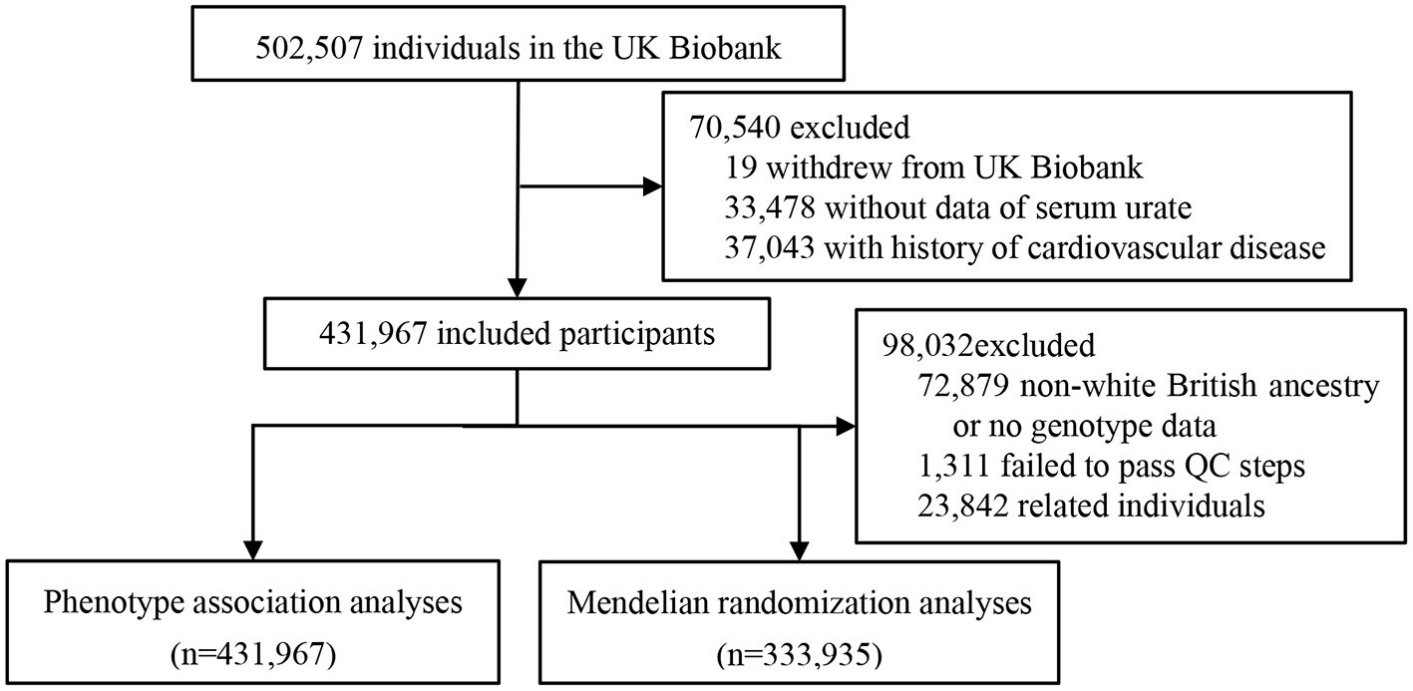
Flow chart for study population selection.

We conducted a cohort analysis including all individuals from UK Biobank. After exclusion of individuals who had withdrawn their data (*n* = 19), without serum urate test (*n* = 33,478), and with a history of CVD at recruitment (*n* = 37,043), our analytic population comprised of 431,967 eligible participants (Figure 1).

At baseline, all participants were categorized as gout, hyperuricemia, and normal groups, and followed up from the recruitment date. Specifically, the level of serum urate >400 μmol/L for males or >360 μmol/L for females was considered as hyperuricemia, based on baseline blood assay test. Individuals with a self-reported medical diagnosis of gout at baseline were assigned into the gout group. Furthermore, individuals (i.e., those in hyperuricemia and normal groups) received a primary diagnosis of gout from inpatient or primary care data according to International Classification of Diseases-10th (ICD-10: M10) during follow-up, were further moved to gout group from the date of gout diagnosis.

All participants were followed until a diagnosis of CVD, death, or the end of study (31st December 2019), whichever occurred first. CVD during follow-up were ascertained by a primary diagnosis of CVD (ICD-10: I00-I70, I730, and I74) in inpatient data, or a death with CVD as the underlying cause from mortality data. In sub-analysis, we studied six major subtypes of CVD (including ischemic heart disease, cerebrovascular disease, emboli/thrombosis, hypertensive disease, heart failure, and arrhythmia/conduction disorder, Supplementary Table 1).

#### Individual-Level Genotyping Data for MR Analysis and Single Nucleotide Polymorphisms Selection

To estimate the causal effect of hyperuricemia or gout on CVD, we conducted MR analysis using imputed genotyping data of 359,088 White British UK Biobank participants. Based on standard genome-wide association study (GWAS) quality control, we first excluded 1,311 individuals who were outliers based on a variant call <98% and 23,842 shared relatedness indicated by a kinship coefficient >0.0884. Ultimately, the analytic dataset contained a total of 7,134,341 variants for 333,935 participants (Figure 1).

We identified 114 independent SNPs associated with hyperuricemia (*p* < 5 × 10^−8^) based on a GWAS study of 288,649 participants of European ancestry (19), among which 96 SNPs were available in our analytic dataset. Particularly, to distinguish the genetic influence of hyperuricemia from that of gout, we further excluded 15 SNPs associated with gout in previous report (19), leaving 81 independent SNPs as instrumental variables for hyperuricemia (explained 1.14% of the variance in hyperuricemia, with corresponding F statistic of 41, list see Supplementary Table 2). Likewise, 92 independent SNPs significantly associated with gout (*p* <5 × 10^−8^) were retrieved from summary GWAS data (19). After removing 86 SNPs that also associated with hyperuricemia [*p* < 5.34 × 10^−4^ (0.05/92)], six SNPs remained as instrumental variables for gout (explained 0.08% of the variance in gout, with corresponding F statistic of 36, list see Supplementary Table 3).

Participants of UK Biobank have signed an informed consent before data collection. The UK Biobank has full ethical approval from the NHS National Research Ethics Service (16/NW/0274), and this study was approved by the biomedical research ethics committee of West China Hospital (2019.1171).

### Statistical Analysis

#### Cohort Analysis on the Phenotypic Association Between Hyperuricemia/Gout and Subsequent CVD

We used Cox regression to assess the associations of hyperuricemia and gout with risk of CVD, presenting as hazard ratios (HRs) with their 95% confidence intervals (CIs). We first estimated the overall associations of hyperuricemia and gout with any CVD, by comparing the CVD risk in the exposed groups to that in unexposed group. Then, such assessments were done for different subtypes of CVD. In all Cox models, we adjusted for sex (female/male), age at follow-up (as continuous variable), ethnicity (White, non-White, or unknown), smoking, alcohol drinking (never, previous, current, or unknown), tea/coffee intake (for each, <2/2–3/4–5/≥6 cups/day, or unknown), physical activity (low, moderate, or high), intake of fish oil and vitamin C/D/E supplementation (for each, yes/no, or unknown), intake of fresh fruit/vegetable (for each, <2/2–2.9/3–3.9/≥4 serving/day, or unknown), intake of processed meat/cheese (for each, never/ <1/1–/≥2 times/week, or unknown), Charlson Comorbidity Index (as continuous variable), and self-reported family history of CVD (yes/no).

#### MR Analysis for Inferring Causal Relationships

In one-sample MR analysis, we used the inverse-variance weighted (IVW) method to pool the individual effect of each eligible SNP in UK Biobank genetic dataset. Specifically, the effect of each genetic instrument on hyperuricemia (51,200 cases and 282,735 controls), gout (9,855 cases and 324,080 controls), and CVD (32,222 cases and 301,713 controls) was assessed by logistic regression model, adjusting for sex, age, genotyping array, and 5 PCs, respectively. Then the causal estimates from multiple SNPs were combined using the inverse square of the standard error for CVD as weight. Besides, as the IVW approach assumed no horizontal pleiotropy (20), we evaluated the presence of horizontal pleiotropy by MR-PRESSO global test (21), and excluded potential outlier SNPs (*p* < 0.05) to correct estimations by MR-PRESSO Outlier-corrected methods.

In two-sample MR analysis, the effect of each genetic instrument on serum urate or gout was obtained from public available summary GWAS data (19), whereas on the CVD was generated from data of White British UK Biobank participants (32,222 cases and 301,713 controls) by logistic regression, adjusting covariates mentioned above.

#### Sensitivity Analysis

To demonstrate the validity of the observed phenotypic associations among individuals involved in the MR analysis, we repeated the cohort analysis among eligible White British UK Biobank participants (*n* = 333,935). To further confirm the differential effects of hyperuricemia and gout on CVD development (i.e., requiring a certain time interval from the studied exposure condition to outcome), as well as to reduce the potential reverse causality, we did a sensitivity analysis by excluding the first 2 years of observation and outcomes detected during this period in each group. Additionally, in order to test the robustness of our analyses to the choice of genetic instruments, we performed 10 times one-sample MR analysis by excluding a randomly selected 10% SNPs from the genetic instrument set at a time, to leave out a subset of the selected variants (21). To further release the concern about possible horizontal pleiotropy of selected genetic instruments, in addition to use MR-PRESSO global test in the main analyses, in a sensitivity analysis, we repeated the MR analyses by additionally excluding SNPs that reported to be also associated with other traits in GWAS Catalog database (i.e., focusing on SNPs with exclusive association with urate or gout, see Supplementary Tables 2, 3).

Statistical analysis was conducted using R, version 4.0.2 (R Project for Statistical Computing). A 2-sided *p* < 0.05 was considered statistically significant.

## Results

### The Phenotypic Association Between Hyperuricemia/Gout and Subsequent CVDs

During a mean follow-up of 10.20 years, 62,752 individuals were into hyperuricemia group, and 12,508 individuals were diagnosed as gout and into group. A higher proportion of both individuals with hyperuricemia and gout was male (66.40 and 84.79%, respectively, compared to 39.32% in normal group, Table 1). No difference was noticed for life style and diet habit among individuals with hyperuricemia and gout, compared with normal group (Table 1).

**Table 1:** Baseline characteristics of study participants in a cohort study of 431,967 individuals from UK Biobank.

**Characteristics**	**Normal group^**a**^ (*N* = 361,199)**	**Hyperuricemia^**b**^ (*N* = 62,752)**	**Gout^**c**^** **(*N* = 12,508)**
Age at follow-up, years, mean (SD)^d^	56.4 (8.11)	57.7 (7.93)	61.0 (7.62)
**Sex**, ***N*** **(%)**^**e**^			
Female	219,164 (60.68)	21,082 (33.60)	1,902 (15.21)
Male	142,035 (39.32)	41,670 (66.40)	10,606 (84.79)
**Race/ethnicity**, ***N*** **(%)**			
White	341,332 (94.50)	59,010 (94.04)	11,900 (95.14)
Non-white	18,711 (5.18)	3,486 (5.56)	567 (4.53)
Unknown	1,156 (0.32)	256 (0.41)	41 (0.33)
**Smoking status**, ***N*** **(%)**			
Never	205,977 (57.03)	30,903 (49.25)	5,609 (44.84)
Previous	116,129 (32.15)	25,557 (40.73)	5,682 (45.43)
Current	37,875 (10.49)	6,034 (9.62)	1,170 (9.35)
Unknown	1,218 (0.34)	258 (0.41)	47 (0.38)
**Alcohol status**, ***N*** **(%)**			
Never	15,990 (4.43)	2,210 (3.52)	273 (2.18)
Previous	12,327 (3.41)	1,900 (3.03)	376 (3.01)
Current	332,503 (92.06)	58,566 (93.33)	11,842 (94.68)
Unknown	379 (0.10)	76 (0.12)	17 (0.14)
**Tea intake**^**f**^, ***N*** **(%)**			
<2	94,598 (26.19)	16,794 (26.76)	3,359 (26.85)
2–3	106,018 (29.35)	18,564 (29.58)	3,936 (31.47)
4–5	91,837 (25.43)	15,838 (25.24)	3,103 (24.81)
≥6	68,003 (18.83)	11,418 (18.20)	2,084 (16.66)
Unknown	743 (0.21)	138 (0.22)	26 (0.21)
**Coffee intake**^**f**^, ***N*** **(%)**			
<2	176,310 (48.81)	31,718 (50.55)	6,642 (53.10)
2–3	113,234 (31.35)	18,891 (30.10)	3,739 (29.89)
4–5	48,564 (13.45)	8,434 (13.44)	1,467 (11.73)
≥6	22,303 (6.17)	3,548 (5.65)	638 (5.10)
Unknown	788 (0.22)	161 (0.26)	22 (0.18)
**Vitamins C**, ***N*** **(%)**			
Yes	32,651 (9.04)	4,858 (7.74)	961 (7.68)
No	327,150 (90.57)	57,577 (91.75)	11,475 (91.74)
Unknown	1,398 (0.39)	317 (0.51)	72 (0.58)
**Vitamins D**, ***N*** **(%)**			
Yes	15,291 (4.23)	1,928 (3.07)	378 (3.02)
No	344,510 (95.38)	60,507 (96.42)	12,058 (96.40)
Unknown	1,398 (0.39)	317 (0.51)	72 (0.58)
**Vitamins E**, ***N*** **(%)**			
Yes	11,427 (3.16)	1,548 (2.47)	322 (2.57)
No	348,374 (96.45)	60,887 (97.03)	12,114 (96.85)
Unknown	1,398 (0.39)	317 (0.51)	72 (0.58)
**Fish oil supplementation**, ***N*** **(%)**			
Yes	114,246 (31.63)	18,605 (29.65)	3,912 (31.28)
No	246,225 (68.17)	43,999 (70.12)	8,576 (68.56)
Unknown	728 (0.20)	148 (0.24)	20 (0.16)
**Fruit intake**^**g**^, ***N*** **(%)**,			
<2	114,671 (31.75)	24,102 (38.41)	4,779 (38.21)
2-	92,293 (25.55)	15,539 (24.76)	3,103 (24.81)
3-	71,417 (19.77)	11,097 (17.68)	2,157 (17.24)
≥4	82,116 (22.73)	11,852 (18.89)	2,439 (19.50)
Unknown	702 (0.19)	162 (0.26)	30 (0.24)
**Vegetable intake**^**g**^, ***N*** **(%)**			
<2	124,600 (34.50)	22,550 (35.94)	4,533 (36.24)
2-	121,702 (33.69)	20,498 (32.67)	4,011 (32.07)
3-	63,744 (17.65)	10,849 (17.29)	2,148 (17.17)
≥4	48,963 (13.56)	8,327 (13.27)	1,715 (13.71)
Unknown	2,190 (0.61)	528 (0.84)	101 (0.81)
**Process meat**^**h**^, ***N*** **(%)**			
Never	37,132 (10.28)	3,357 (5.35)	509 (4.07)
<1	114,863 (31.80)	15,740 (25.08)	2,789 (22.30)
1-	104,249 (28.86)	18,870 (30.07)	3,675 (29.38)
≥2	104,207 (28.85)	24,625 (39.24)	5,511 (44.06)
Unknown	748 (0.21)	160 (0.25)	24 (0.19)
**Cheese** ^**h**^ **, N (%)**			
Never	9,048 (2.50)	1,798 (2.87)	380 (3.04)
<1	59,784 (16.55)	10,449 (16.65)	2,043 (16.33)
1-	74,227 (20.55)	13,551 (21.59)	2,786 (22.27)
≥2	209,267 (57.94)	35,168 (56.04)	6,899 (55.16)
Unknown	8,873 (2.46)	1,786 (2.85)	400 (3.20)
**Physical activity**, ***N*** **(%)**			
Low	71,521 (19.80)	14,416 (22.97)	2,868 (22.93)
Moderate	156,282 (43.27)	27,165 (43.29)	5,347 (42.75)
High	133,396 (36.93)	21,171 (33.74)	4,293 (34.32)
**Family history of CVD**, ***N*** **(%)**			
Yes	198,953 (55.08)	35,406 (56.42)	7,155 (57.20)
No	162,246 (44.92)	27,346 (43.58)	5,353 (42.80)
CCI score, mean (SD)	0.184 (0.783)	0.247 (0.915)	0.445 (1.22)

a *Normal group: Individuals were not defined as hyperuricemia and gout at baseline and during follow-up*.

b *Hyperuricemia: The level of serum urate at baseline, >400 μmol/L (6.8 mg/dL, for males) or >360 μmol/L (6 mg/dL, for females)*.

c *Gout: Either diagnosis in UK Biobank inpatient or primary care data (ICD-10: M10), or self-reported a medical diagnosis of gout, at baseline or during the follow-up*.

d *Mean (SD): Mean (Standard Deviation)*.

e *N (%): Number (%)*.

f *Measured as cups per day*.

g *Servings/day: Two heaped tablespoons of vegetables were counted as a serving; two pieces of fresh fruit or four pieces of dried fruit were counted as a serving*.

h *Measured as times per week*.

Compared to individuals without hyperuricemia and gout throughout the study period, patients with hyperuricemia at baseline had higher incidence of any CVDs during follow-up [incidence rate (IR) = 13.54 vs. 8.15), which corresponded to a HR of 1.33 (95% CI: 1.29–1.36, Table 2). Notably, patients exposed to a diagnosis of gout experienced even higher elevation in CVD risk (IR = 19.53; HR = 1.54, 95% CI: 1.48–1.62, Table 2).

**Table 2:** Incidence rate and hazard ratios of any cardiovascular disease (CVD)^a^ among patients with studied hyperuricemia or gout when compared with patients without hyperuricemia and gout.

	**Hyperuricemia** ^**b**^		**Gout** ^**c**^	
**Any CVD**	**Incidence rate^**d**^**	**HR (95% CI)^**e**^**	**Incidence rate**	**HR (95% CI)**
Unexposed group	25,852/3,117.77 (8.29)	Ref	2,5852/3,117.77 (8.29)	Ref
Exposed group	6,965/509 (13.68)	1.32 (1.28–1.35)	1,731/89.08 (19.43)	1.51 (1.44–1.59)

a *A primary diagnosis of CVD in UK Biobank inpatient data, or a death with CVD as the underlying cause, according to UK Biobank mortality data (ICD-10: I00-I70, I730, and I74)*.

b *Hyperuricemia: The level of serum urate at baseline, >400 μmol/L (6.8 mg/dL, for males) or >360 μmol/L (6 mg/dL, for females)*.

c *Gout: Either diagnosis in UK Biobank inpatient or primary care data (ICD-10: M10), or self-reported a medical diagnosis of gout, at baseline or during the follow-up*.

d *Incidence rate was measured as No. of Cases/No. of Accumulated Person-Years ×1,000 (Incidence Rate/1,000 Person-Years)*.

e *HR, hazard ratio; CI, confidence interval; models adjusted for sex, age at follow-up, ethnicity, smoking, alcohol drinking, tea intake, coffee intake, physical activity, intake of fish oil supplementation, intake of vitamin C/D/E supplementation, intake of fresh fruit/vegetable, intake of processed meat/cheese, Charlson Comorbidity Index, and self-reported family history of CVD; individuals without hyperuricemia and gout were used*.

**Figure 2:**
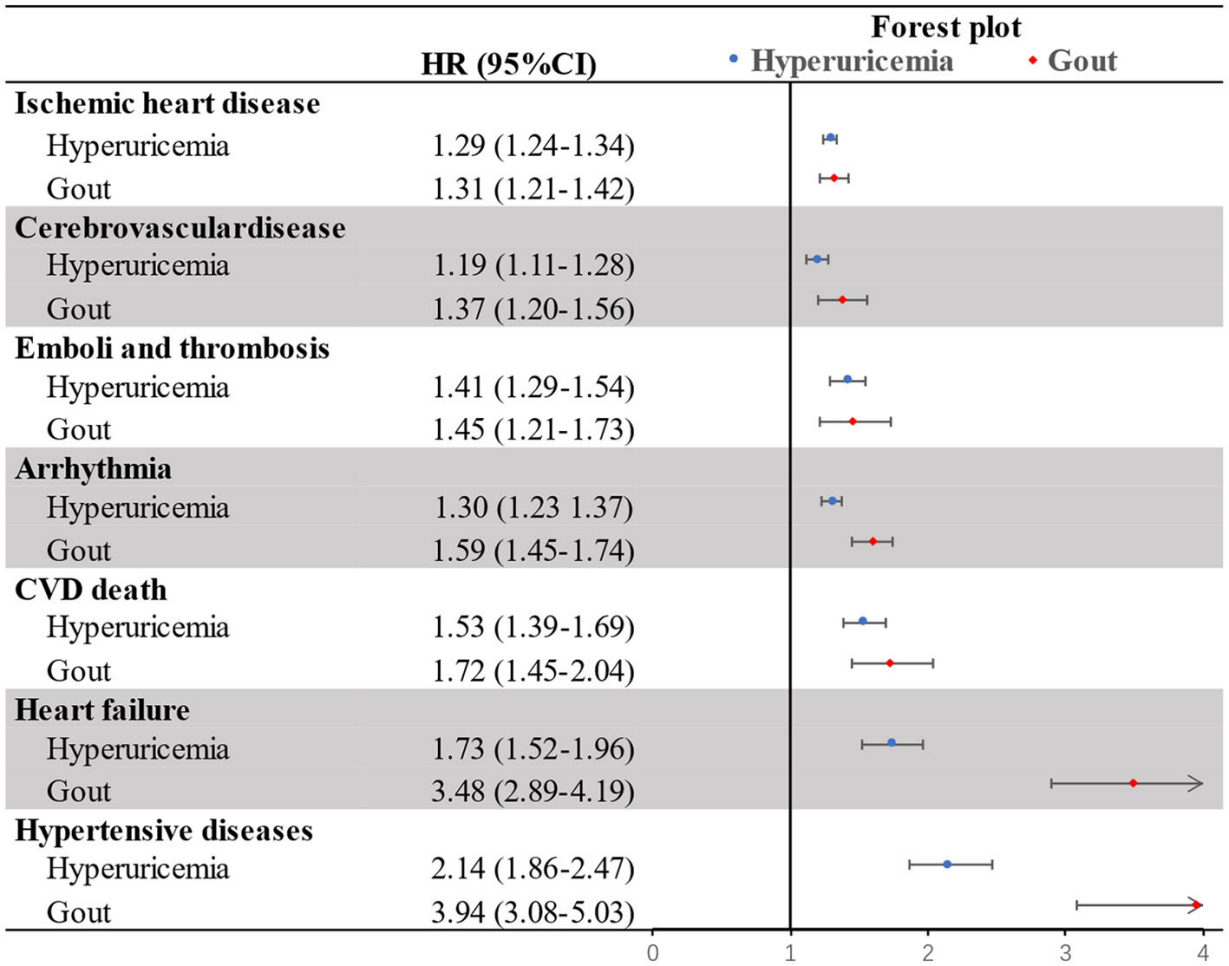
Hazard ratios of specific cardiovascular diseases (CVDs) among patients with studied hyperuricemia or gout when compared with patients without hyperuricemia and gout.

The sub-analysis for specific CVD revealed the increased risk associated with hyperuricemia/gout generally existed for all studied subtypes of CVD, with the top estimates always observed for hypertensive diseases (Figure 2). Furthermore, while the level of risk increase was comparable between hyperuricemia and gout for most studied CVDs, we observed that patients with gout tended to have higher risk for heart failure and hypertensive diseases, compared to individuals with hyperuricemia (Figure 2).

In the sensitivity analysis restricting to eligible White British UK Biobank participants, largely identical estimates were obtained, compared to the results of the main analysis (Supplementary Table 4; Supplementary Figure 1). Similarly, these observed associations stayed robust after excluding the first 2 years of follow-up (Supplementary Table 5).

### Causal Relationships Between Hyperuricemia/Gout and CVDs

The application of MR-PRESSO global test identified only one outlier SNP (rs10857147) for hyperuricemia, while indicated no violation of horizontal pleiotropy assumption for selected genetically instrumental variants for gout (*p* > 0.05). Utilizing the IVM methods, we found significantly positive association between genetic liability for serum urate level and CVDs in both one-sample (OR = 1.06, 95% CI: 1.02–1.11, *p* < 0.05) and two-sample (OR = 1.09, 95% CI: 1.03–1.16, *p* < 0.05, Table 3) MR analysis. Further outlier-correction did not materially change the OR estimates (OR = 1.07, 95% CI: 1.02–1.11, *p* < 0.05, and OR = 1.10, 95% CI: 1.04–1.16, *p* < 0.05 for one- and two-sample MR, respectively). However, using SNPs specifically associated with gout but not serum urate, we found on association between genetic liability for gout and CVD in either one-sample (OR = 0.89, 95% CI: 0.79–1.01, *p* = 0.078) or two-sample (OR = 0.92, 95% CI: 0.82–1.21, *p* = 0.192, Table 3) MR analysis.

**Table 3:** Association of hyperuricemia and gout with risk of cardiovascular disease (CVD)^a^ using Mendelian Randomization (MR) analyses.

	**One-sample MR analysis**		**Two-sample MR analysis**	
**Exposure**	**OR (95% CI)^**d**^**	***P***	**OR (95% CI)^**d**^**	***P***
Hyperuricemia^b^	1.06 (1.02–1.11)	0.009	1.09 (1.03–1.16)	0.005
Gout^c^	0.89 (0.79–1.01)	0.078	0.92 (0.82–1.21)	0.192

a *A primary diagnosis of CVD in UK Biobank inpatient data, or a death with CVD as the underlying cause, according to UK Biobank mortality data (ICD-10: I00-I70, I730, and I74)*.

b *Hyperuricemia: 81 independent and not related to gout SNPs derived from published GWAS data were used as instrumental variables to infer causality*.

c *Gout: 6 SNPs were used instrumental variables, which was not related to hyperuricemia*.

d *OR, Odds ratio; CI, confidence interval; models adjusted for sex, age, genotyping array, and 5 PCs*.

In the sensitivity analysis, exclusion of 10% selected SNPs led to similar estimates as the main analysis, where we also observed significantly positive effect of genetic liability for serum urate level on CVD in one-sample MR analysis (Supplementary Figure 2). Again, nine of 10 times one-sample MR sensitivity analysis showed no causal association for gout (Supplementary Figure 2). Also, by additionally removing SNPs associated with other traits (e.g., weight, chronic kidney disease, etc., leaving 44 SNPs for hyperuricemia and five SNPs for gout), the estimates obtained from the MR analyses were not modified largely (Supplementary Table 6).

## Discussion

Based on this cohort study of over 460,000 participants, our results concluded that both individuals with hyperuricemia detected through screening and those got a clinical diagnosis of gout experienced increased risk of developing multiple types of CVD and CVD death. Particularly, together with the special efforts on distinguishing the effect of asymptomatic hyperuricemia from that of gout on CVD outcomes in the cohort analysis, our selection of instrumental genetic variables in MR analysis also aimed separate SNPs genetically association with hyperuricemia from those with a clinical diagnosis of gout. Consequently, based on results of both one-sample and two-sample MR analyses, we found only supportive evidence on the causal relationship between hyperuricemia and CVD, while the association between genetically determined gout on CVD outcomes seems to be not causal.

Our findings of increased CVD risk among individuals with hyperuricemia and gout is consistent with previous studies. A study with 16.4 years follow-up, included 5,926 subjects who had serum urate level measurements at baseline, found that increased serum urate levels had a positive relationship to CVD mortality in men and women, among black and white persons (22). Furthermore, using data on a clinic-based cohort of 706 patients with gout, the presence of subcutaneous tophi and high baseline serum urate level were found as independent risk factor for increased CVD mortality (23). Importantly, such risk increase was in parallel with serum urate levels (23), and with increasing severity of gout (24). While most of these analyses were conducted when serum urate level and gout status was measured at the same time, our results add existing literature by demonstrating the independency of screening-identified serum urate and gout diagnosis on CVD risk, using longitudinal data.

The causality between hyperuricemia with CVD remains inclusive. Li et al. reported a potentially causal linkage between genetic determined higher serum urate level and increased risk of hypertensive disease, including essential hypertension and myocardial infarction, which however might be attributed to the pleiotropic effect of multiple instruments and unbalanced pleiotropy (14). A more recent meta-analysis of 58 studies suggested a causal role of urate in the development of coronary heart disease by MR analysis, while the possibility of unbalanced pleiotropy which have inflated the estimates was also noted (25). In contrast, null results were also described in other studies where no evidence was found for a causal relationship between urate and CHD and heart failure (26). Our attempts of examining the causal effect of serum urate level or gout specifically on CVDs are novel. Importantly, as the results support an association between genetic liability for serum urate level, but not gout, and CVDs, our findings imply that the presence of hyperuricemia can increase the risk of CVDs, while the further enhanced risk elevation for individuals with gout (i.e., the increasing effect from hyperuricemia-gout on CVD) may due to either higher level and prolonged effect of serum urate, or joint impacts of between serum urate and environmental risk factors (e.g., obesity, reduced physical activity, reduced fish intake, etc.) on CVDs among such a population.

Although the detailed mechanisms remain inconclusive, several potential pathways have been proposed for the explanation of the association between hyperuricemia and increased CVDs. First, both experimental and human studies demonstrate that the increase of serum urate may induce endothelial dysfunction through increased oxidative stress and inflammation (27). Also, uric acid can stimulate vascular smooth muscle cell proliferation and oxidative stress possibly through the vascular renin-angiotensin system (28), which further play a central role in the development of various CVDs. Furthermore, hyperuricemia has been noted as a cause of arteriolar disease in kidney by impairing autoregulatory response (29); and the impaired autoregulatory response of the cerebral arterioles was closely associated with increased risk for stroke (30). Instead, biological evidence linking gout with CVD is limited, which mainly focus on the inflammation status (e.g., the overproduction of proinflammatory cytokines) in joints (27, 31). However, as the precipitation and deposition of uric acid crystals in synovial fluid and tissues is a well-identified consequence of hyperuricemia. It keeps unknown whether gout can increase the risk of CVD outside of the uric acid pathway. Here, our MR analysis indicates the stronger effect of gout on CVDs, relative to hyperuricemia on CVD in phenotypic analysis, might attribute to some important lifestyle factors that generally observed among the gout patients, such as the incapability of physical activity and reduced fish intake. Also, some comorbidities of gout, such as hyperlipidemia, obesity, and diabetes, are also identified as risk factors for CVD (32). Collectively, although need further verification, this finding highlights the necessities and importance of exploring feasible interventions on comorbid conditions and lifestyles among individuals with gout, in the terms of CVD prevention.

Our present study has several strengths. First, the application of UK Biobank, where the combination use of enriched phenotypic data, complete medical follow-up data, and individual-level genotyping data is possible, enabled a comprehensive assessment on the temporal relationship, as well as its underlying mechanisms, of hyperuricemia, gout, and CVD. Second, because we separated the genetic variants for hyperuricemia from those for gout specifically as instrumental variables in MR analysis, our results add to the existing literature by elucidating the effects of other factors that associated with gout on CVDs, in addition to the uric acid pathway.

Notable limitations include the small number of genetic instruments for both serum urate and gout in MR analysis. Therefore, future studies on causal assessment are needed for verification of our results, with ideally improved knowledge on genetic determinants on these traits. In addition, although the MR-PRESSO global test indicated no violation of horizontal pleiotropy and the sensitivity MR analyses where SNPs with reported association with other traits were additionally removed showed similar estimates, the concern that these genetic variants may the outcome through other pathways than the studied exposures cannot be completely addressed. However, the robustness of our results on the choose of genetic instruments has been partly demonstrated by the similar estimates observed in sensitivity analyses where we repeated 10 times of the analysis by randomly removing 10% of analyzed SNPs at each time. Moreover, our measurement on serum urate level was merely based on the blood tests at recruitment. Further studies, with ideally dynamic surveillance on serum urate, are warranted to provide more accurate assessment on the association between asymptotic hyperuricemia and CVD. Nevertheless, as a causal link was suggested between serum urate level and CVD in our analysis, the timely intervention on hyperuricemia may need, regardless of the presence of clinical symptoms nor the diagnosis of gout. Last, as the primary care data covered only 45% of UK Biobank patients, a part of patients with mild to moderate gout may be missed in our analysis. Also, as the absence of information on the use of uric acid lowering therapy among our participants, there are possibilities of misclassification, which renders unclear impacts on our estimates.

In conclusion, based on a longitudinal cohort study in UK Biobank, our results demonstrated a reliable association between hyperuricemia/gout on various types of CVD. Furthermore, as the MR analyses where we applied exclusive genetic variants for hyperuricemia and for gout suggest a causal effect of serum urate level, but not gout, on CVDs, our results indicate the possible effects of other gout-associated factors on the development of CVDs, in addition to the uric acid pathway, underscoring the exploration of feasible interventions on comorbid conditions and lifestyles among individuals with gout, for CVD prevention.

## Data Availability Statement

Data from the UK Biobank (http://www.ukbiobank.ac.uk/) are available to all researchers upon making an application.

## Ethics Statement

Participants of UK Biobank have signed an informed consent before data collection. The UK Biobank has full ethical approval from the NHS National Research Ethics Service (16/NW/0274), and this study was approved by the biomedical research ethics committee of West China Hospital (2019.1171).

## Author Contributions

JZ and HS were responsible for the study's concept and design. HY, WC, YH, YS, ZY, and YQ did the data and project management. YZ and HZ did the data cleaning and analysis. JZ, YZ, HZ, HY, WC, and HS interpreted the data. JZ, YZ, HZ, and HS drafted the manuscript. All the authors approved the final manuscript as submitted and agree to be accountable for all aspects of the work.

## Funding

This work was supported by the National Science Foundation of China (No. 81971262 to HS), 1.3.5 project for disciplines of excellence, West China Hospital, Sichuan University (No. ZYYC21005 to HS), and Science and Technology Department of Sichuan Province (Nos. 2021YFS0180 to JZ and 2020YFS0575 to HS).

## Conflict of Interest

The authors declare that the research was conducted in the absence of any commercial or financial relationships that could be construed as a potential conflict of interest.

## Publisher's Note

All claims expressed in this article are solely those of the authors and do not necessarily represent those of their affiliated organizations, or those of the publisher, the editors and the reviewers. Any product that may be evaluated in this article, or claim that may be made by its manufacturer, is not guaranteed or endorsed by the publisher.
