# Supplementary material for: The Association of Hyperuricemia and Gout With the Risk of Cardiovascular Diseases: A Cohort and Mendelian Randomization Study in UK Biobank
Source: Front Med (Lausanne). 2022 Mar 23;8:817150. doi: 10.3389/fmed.2021.817150 (PMC8985123; doi:10.3389/fmed.2021.817150)
Supplement: Supplementary file 1 [file Data_Sheet_1.docx]

| Supplementary Table 1 International Classification of Diseases (ICD) codes for outcome identifications | |
| --- | --- |
| Diagnostic groups | ICD-10 |
| Any cardiovascular disease | I00-I70, I730, I74 |
| Ischemic heart disease | I20, I21, I22, I23, I24, I25 (excl. I25.5) |
| Cerebrovascular disease | I60-I69 |
| Emboli and thrombosis | I74, I26 |
| Heart failure | I50, I25.5, I42.0, I42.8, I42.9 |
| Hypertensive diseases | I10- I15 |
| Arrhythmia/conduction disorder | I44-I49 |
| Cardiovascular disease death | I00-I70, I730, I74 |

| Supplementary Table2 Summary of index SNPs identified in previous urate GWAS used as genetic instruments for hyperuricemia | | | | | | | | | |
| --- | --- | --- | --- | --- | --- | --- | --- | --- | --- |
| SNP | Chromo-some | Position (b37) | Coded allele | Non-coded allele | Coded allele freq | Effect | SE | p-value | Reported traits in GWAS Catalog |
| rs4646068 | 1 | 15,828,704 | t | c | 0.692 | 0.024 | 0.004 | 7.71E-09 | [urate measurement](https://www.ebi.ac.uk/gwas/efotraits/EFO_0004531) |
| rs139428292 | 1 | 145,507,646 | a | g | 0.027 | -0.073 | 0.013 | 4.91E-08 | [urate measurement](https://www.ebi.ac.uk/gwas/efotraits/EFO_0004531) |
| rs11204701 | 1 | 150,662,179 | a | t | 0.779 | -0.036 | 0.005 | 1.05E-14 | [urate measurement](https://www.ebi.ac.uk/gwas/efotraits/EFO_0004531) |
| rs2760215 | 1 | 163,675,883 | t | c | 0.503 | -0.025 | 0.004 | 5.81E-11 | [urate measurement](https://www.ebi.ac.uk/gwas/efotraits/EFO_0004531) |
| rs12037861 | 1 | 221,038,177 | a | t | 0.704 | 0.023 | 0.004 | 3.39E-08 | u[rate measurement](https://www.ebi.ac.uk/gwas/efotraits/EFO_0004531) |
| rs72782806 | 2 | 15,788,511 | a | g | 0.26 | 0.025 | 0.004 | 8.12E-09 | [urate measurement](https://www.ebi.ac.uk/gwas/efotraits/EFO_0004531) |
| rs12472381 | 2 | 59,321,225 | a | g | 0.39 | 0.022 | 0.004 | 1.80E-08 | [urate measurement](https://www.ebi.ac.uk/gwas/efotraits/EFO_0004531) |
| rs12987661 | 2 | 69,813,458 | t | c | 0.866 | 0.041 | 0.006 | 1.44E-12 | [urate measurement](https://www.ebi.ac.uk/gwas/efotraits/EFO_0004531); [hemoglobin measurement](https://www.ebi.ac.uk/gwas/efotraits/EFO_0004509); [hematocrit](https://www.ebi.ac.uk/gwas/efotraits/EFO_0004348); [dihydroxy docosatrienoic acid measurement](https://www.ebi.ac.uk/gwas/efotraits/EFO_0005275); [erythrocyte count](https://www.ebi.ac.uk/gwas/efotraits/EFO_0004305); [uric acid measurement](https://www.ebi.ac.uk/gwas/efotraits/EFO_0004761) |
| rs17050272 | 2 | 121,306,440 | a | g | 0.421 | 0.032 | 0.004 | 1.57E-15 | [urate measurement](https://www.ebi.ac.uk/gwas/efotraits/EFO_0004531); [low density lipoprotein cholesterol measurement](https://www.ebi.ac.uk/gwas/efotraits/EFO_0004611); [glomerular filtration rate](https://www.ebi.ac.uk/gwas/efotraits/EFO_0005208); [sex hormone-binding globulin measurement](https://www.ebi.ac.uk/gwas/efotraits/EFO_0004696);[serum gamma-glutamyl transferase measurement](https://www.ebi.ac.uk/gwas/efotraits/EFO_0004532); [uric acid measurement](https://www.ebi.ac.uk/gwas/efotraits/EFO_0004761): [total cholesterol measurement](https://www.ebi.ac.uk/gwas/efotraits/EFO_0004574);[creatinine measurement](https://www.ebi.ac.uk/gwas/efotraits/EFO_0004518); [blood urea nitrogen measurement](https://www.ebi.ac.uk/gwas/efotraits/EFO_0004741); [apolipoprotein B measurement](https://www.ebi.ac.uk/gwas/efotraits/EFO_0004615) |
| rs11683692 | 2 | 145,509,615 | t | c | 0.944 | -0.048 | 0.008 | 1.32E-08 | urate measurement |
| rs1234413 | 2 | 148,844,369 | t | c | 0.442 | -0.022 | 0.004 | 7.08E-09 | [urate measurement](https://www.ebi.ac.uk/gwas/efotraits/EFO_0004531) |
| rs9287911 | 2 | 170,037,294 | a | t | 0.25 | 0.038 | 0.004 | 1.13E-17 | [urate measurement](https://www.ebi.ac.uk/gwas/efotraits/EFO_0004531) |
| rs187355703 | 2 | 176,993,583 | c | g | 0.975 | -0.086 | 0.013 | 2.70E-11 | [glomerular filtration rate](https://www.ebi.ac.uk/gwas/efotraits/EFO_0005208); [blood urea nitrogen measurement](https://www.ebi.ac.uk/gwas/efotraits/EFO_0004741); [urate measurement](https://www.ebi.ac.uk/gwas/efotraits/EFO_0004531); [chronic kidney disease](https://www.ebi.ac.uk/gwas/efotraits/EFO_0003884); [creatinine measurement](https://www.ebi.ac.uk/gwas/efotraits/EFO_0004518); [cystatin c measurement](https://www.ebi.ac.uk/gwas/efotraits/EFO_0004617); [serum urea measurement](https://www.ebi.ac.uk/gwas/efotraits/EFO_0009795); [uric acid measurement](https://www.ebi.ac.uk/gwas/efotraits/EFO_0004761) |
| rs1047891 | 2 | 211,540,507 | a | c | 0.311 | -0.024 | 0.004 | 2.09E-08 | [serum metabolite measurement](https://www.ebi.ac.uk/gwas/efotraits/EFO_0005653); [urinary metabolite measurement](https://www.ebi.ac.uk/gwas/efotraits/EFO_0005116); [glomerular filtration rate](https://www.ebi.ac.uk/gwas/efotraits/EFO_0005208); [mean corpuscular volume](https://www.ebi.ac.uk/gwas/efotraits/EFO_0004526); [high density lipoprotein cholesterol measurement](https://www.ebi.ac.uk/gwas/efotraits/EFO_0004612); [mean corpuscular hemoglobin](https://www.ebi.ac.uk/gwas/efotraits/EFO_0004527), etc. |
| rs9288447 | 2 | 213,083,638 | t | c | 0.546 | -0.023 | 0.004 | 3.27E-09 | [urate measurement](https://www.ebi.ac.uk/gwas/efotraits/EFO_0004531) |
| rs2581817 | 3 | 53,071,797 | c | g | 0.42 | 0.048 | 0.004 | 4.87E-35 | urate measurement; gout |
| rs7640441 | 3 | 125,118,082 | a | c | 0.246 | -0.028 | 0.005 | 1.26E-09 | [urate measurement](https://www.ebi.ac.uk/gwas/efotraits/EFO_0004531) |
| rs11718633 | 3 | 126,012,421 | t | c | 0.198 | -0.028 | 0.005 | 7.05E-09 | [urate measurement](https://www.ebi.ac.uk/gwas/efotraits/EFO_0004531) |
| rs80120242 | 3 | 132,235,344 | a | t | 0.947 | -0.062 | 0.01 | 1.87E-09 | [urate measurement](https://www.ebi.ac.uk/gwas/efotraits/EFO_0004531) |
| rs62294340 | 3 | 169,155,476 | a | g | 0.364 | -0.022 | 0.004 | 5.00E-08 | [urate measurement](https://www.ebi.ac.uk/gwas/efotraits/EFO_0004531); [uric acid measurement](https://www.ebi.ac.uk/gwas/efotraits/EFO_0004761) |
| rs62286563 | 4 | 10,122,665 | t | g | 0.979 | -0.103 | 0.015 | 1.16E-12 | [urate measurement](https://www.ebi.ac.uk/gwas/efotraits/EFO_0004531) |
| rs73224492 | 4 | 10,440,925 | a | g | 0.875 | -0.094 | 0.006 | 9.00E-59 | [urate measurement](https://www.ebi.ac.uk/gwas/efotraits/EFO_0004531) |
| rs98270 | 4 | 48,019,323 | a | g | 0.362 | 0.022 | 0.004 | 4.21E-08 | [urate measurement](https://www.ebi.ac.uk/gwas/efotraits/EFO_0004531) |
| rs10857147 | 4 | 81,181,072 | a | t | 0.713 | 0.024 | 0.004 | 2.21E-08 | [coronary artery disease](https://www.ebi.ac.uk/gwas/efotraits/EFO_0001645); [urate measurement](https://www.ebi.ac.uk/gwas/efotraits/EFO_0004531); [mean arterial pressure](https://www.ebi.ac.uk/gwas/efotraits/EFO_0006340); measurement |
| rs1481012 | 4 | 89,039,082 | a | g | 0.889 | -0.249 | 0.006 | 0 | [urate measurement](https://www.ebi.ac.uk/gwas/efotraits/EFO_0004531); [tea consumption measurement](https://www.ebi.ac.uk/gwas/efotraits/EFO_0010091); [coffee consumption](https://www.ebi.ac.uk/gwas/efotraits/EFO_0004330); [gout](https://www.ebi.ac.uk/gwas/efotraits/EFO_0004274); [body mass index](https://www.ebi.ac.uk/gwas/efotraits/EFO_0004340) |
| rs1440411 | 4 | 144,158,285 | t | c | 0.571 | -0.028 | 0.004 | 1.08E-12 | [urate measurement](https://www.ebi.ac.uk/gwas/efotraits/EFO_0004531) |
| rs455213 | 5 | 34,660,235 | t | c | 0.543 | -0.027 | 0.004 | 6.05E-12 | [urate measurement](https://www.ebi.ac.uk/gwas/efotraits/EFO_0004531); [red blood cell density measurement](https://www.ebi.ac.uk/gwas/efotraits/EFO_0007978); [erythrocyte count](https://www.ebi.ac.uk/gwas/efotraits/EFO_0004305); [hemoglobin measurement](https://www.ebi.ac.uk/gwas/efotraits/EFO_0004509); [uric acid measurement](https://www.ebi.ac.uk/gwas/efotraits/EFO_0004761) |
| rs76004499 | 5 | 176,705,865 | c | g | 0.972 | -0.074 | 0.013 | 3.27E-08 | [urate measurement](https://www.ebi.ac.uk/gwas/efotraits/EFO_0004531) |
| rs12530084 | 6 | 7,214,676 | t | c | 0.22 | 0.066 | 0.005 | 9.55E-48 | [urate measurement](https://www.ebi.ac.uk/gwas/efotraits/EFO_0004531) |
| rs198851 | 6 | 26,104,632 | t | g | 0.144 | 0.039 | 0.005 | 5.80E-13 | [mean corpuscular hemoglobin](https://www.ebi.ac.uk/gwas/efotraits/EFO_0004527); [mean corpuscular volume](https://www.ebi.ac.uk/gwas/efotraits/EFO_0004526); [reticulocyte count](https://www.ebi.ac.uk/gwas/efotraits/EFO_0007986); [red blood cell distribution width](https://www.ebi.ac.uk/gwas/efotraits/EFO_0005192); [Diuretic use measurement](https://www.ebi.ac.uk/gwas/efotraits/EFO_0009928); [alcohol consumption measurement](https://www.ebi.ac.uk/gwas/efotraits/EFO_0007878), [diastolic blood pressure](https://www.ebi.ac.uk/gwas/efotraits/EFO_0006336) |
| rs742493 | 6 | 40,998,167 | t | c | 0.881 | 0.039 | 0.006 | 9.79E-11 | [urate measurement](https://www.ebi.ac.uk/gwas/efotraits/EFO_0004531); [vitamin D measurement](https://www.ebi.ac.uk/gwas/efotraits/EFO_0004631); [protein measurement](https://www.ebi.ac.uk/gwas/efotraits/EFO_0004747); [uric acid measurement](https://www.ebi.ac.uk/gwas/efotraits/EFO_0004761) |
| rs1574430 | 6 | 43,269,029 | a | c | 0.405 | 0.029 | 0.004 | 2.77E-14 | [urate measurement](https://www.ebi.ac.uk/gwas/efotraits/EFO_0004531) |
| rs10223666 | 6 | 43,805,502 | c | g | 0.704 | 0.046 | 0.004 | 6.62E-28 | [urate measurement](https://www.ebi.ac.uk/gwas/efotraits/EFO_0004531); [BMI-adjusted waist circumference](https://www.ebi.ac.uk/gwas/efotraits/EFO_0007789); [creatinine measurement](https://www.ebi.ac.uk/gwas/efotraits/EFO_0004518); [glomerular filtration rate](https://www.ebi.ac.uk/gwas/efotraits/EFO_0005208); [hypothyroidism](https://www.ebi.ac.uk/gwas/efotraits/EFO_0004705); [glucose measurement](https://www.ebi.ac.uk/gwas/efotraits/EFO_0004468) |
| rs4897160 | 6 | 126,223,944 | a | g | 0.483 | 0.03 | 0.004 | 1.96E-14 | [urate measurement](https://www.ebi.ac.uk/gwas/efotraits/EFO_0004531); [hemoglobin measurement](https://www.ebi.ac.uk/gwas/efotraits/EFO_0004509); [hematocrit](https://www.ebi.ac.uk/gwas/efotraits/EFO_0004348); [uric acid measurement](https://www.ebi.ac.uk/gwas/efotraits/EFO_0004761) |
| rs13226650 | 7 | 73,017,005 | a | g | 0.809 | 0.049 | 0.005 | 1.35E-23 | [urate measurement](https://www.ebi.ac.uk/gwas/efotraits/EFO_0004531); [high density lipoprotein cholesterol measurement](https://www.ebi.ac.uk/gwas/efotraits/EFO_0004612), [sleep duration](https://www.ebi.ac.uk/gwas/efotraits/EFO_0005271); [metabolic syndrome](https://www.ebi.ac.uk/gwas/efotraits/EFO_0000195) |
| rs10480300 | 7 | 151,406,005 | t | c | 0.276 | 0.03 | 0.004 | 4.26E-12 | [urate measurement](https://www.ebi.ac.uk/gwas/efotraits/EFO_0004531); [gout](https://www.ebi.ac.uk/gwas/efotraits/EFO_0004274); [hemoglobin measurement](https://www.ebi.ac.uk/gwas/efotraits/EFO_0004509) |
| rs34861762 | 8 | 23,748,420 | t | c | 0.419 | 0.034 | 0.004 | 3.50E-19 | [urate measurement](https://www.ebi.ac.uk/gwas/efotraits/EFO_0004531); [glomerular filtration rate](https://www.ebi.ac.uk/gwas/efotraits/EFO_0005208); [creatinine measurement](https://www.ebi.ac.uk/gwas/efotraits/EFO_0004518); [chronic kidney disease](https://www.ebi.ac.uk/gwas/efotraits/EFO_0003884) |
| rs2466077 | 8 | 32,432,753 | t | g | 0.533 | -0.022 | 0.004 | 1.78E-08 | [urate measurement](https://www.ebi.ac.uk/gwas/efotraits/EFO_0004531) |
| rs2943539 | 8 | 76,479,839 | t | c | 0.475 | 0.041 | 0.004 | 6.42E-28 | [urate measurement](https://www.ebi.ac.uk/gwas/efotraits/EFO_0004531) |
| rs10956924 | 8 | 95,678,312 | t | c | 0.279 | -0.024 | 0.004 | 1.79E-08 | [urate measurement](https://www.ebi.ac.uk/gwas/efotraits/EFO_0004531) |
| rs10971420 | 9 | 33,125,000 | t | c | 0.688 | 0.031 | 0.004 | 4.14E-14 | [urate measurement](https://www.ebi.ac.uk/gwas/efotraits/EFO_0004531); [serum IgG glycosylation measurement](https://www.ebi.ac.uk/gwas/efotraits/EFO_0005193) |
| rs56106601 | 9 | 130,770,484 | a | c | 0.946 | 0.061 | 0.009 | 2.68E-11 | [urate measurement](https://www.ebi.ac.uk/gwas/efotraits/EFO_0004531) |
| rs9420446 | 10 | 88,880,689 | t | c | 0.137 | -0.038 | 0.006 | 1.13E-11 | [urate measurement](https://www.ebi.ac.uk/gwas/efotraits/EFO_0004531); [erythrocyte count](https://www.ebi.ac.uk/gwas/efotraits/EFO_0004305); [glomerular filtration rate](https://www.ebi.ac.uk/gwas/efotraits/EFO_0005208) |
| rs35198068 | 10 | 114,754,784 | t | c | 0.706 | 0.025 | 0.004 | 5.85E-09 | [urate measurement](https://www.ebi.ac.uk/gwas/efotraits/EFO_0004531); [body mass index](https://www.ebi.ac.uk/gwas/efotraits/EFO_0004340); [sex hormone-binding globulin measurement](https://www.ebi.ac.uk/gwas/efotraits/EFO_0004696); [psoriasis](https://www.ebi.ac.uk/gwas/efotraits/EFO_0000676), [type ii diabetes mellitus](https://www.ebi.ac.uk/gwas/efotraits/EFO_0001360); [body weight](https://www.ebi.ac.uk/gwas/efotraits/EFO_0004338) |
| rs35506085 | 11 | 2,165,576 | a | g | 0.189 | -0.029 | 0.005 | 1.50E-08 | [BMI-adjusted hip circumference](https://www.ebi.ac.uk/gwas/efotraits/EFO_0008039); [lean body mass](https://www.ebi.ac.uk/gwas/efotraits/EFO_0004995); [body height](https://www.ebi.ac.uk/gwas/efotraits/EFO_0004339); [body height](https://www.ebi.ac.uk/gwas/efotraits/EFO_0004339) |
| rs148185902 | 11 | 30,718,534 | a | g | 0.012 | 0.123 | 0.023 | 5.76E-08 | [urate measurement](https://www.ebi.ac.uk/gwas/efotraits/EFO_0004531) |
| rs3925584 | 11 | 30,760,335 | t | c | 0.552 | 0.03 | 0.004 | 1.66E-15 | glomerular filtration rate; creatinine measurement; blood urea nitrogen measurement; [urate measurement](https://www.ebi.ac.uk/gwas/efotraits/EFO_0004531); [chronic kidney disease](https://www.ebi.ac.uk/gwas/efotraits/EFO_0003884); [magnesium measurement](https://www.ebi.ac.uk/gwas/efotraits/EFO_0004845); [diastolic blood pressure](https://www.ebi.ac.uk/gwas/efotraits/EFO_0006336); [systolic blood pressure](https://www.ebi.ac.uk/gwas/efotraits/EFO_0006335) |
| rs71456318 | 11 | 64,332,862 | a | c | 0.484 | 0.079 | 0.004 | 4.41E-92 | urate measurement |
| rs2022051 | 11 | 64,367,589 | a | g | 0.793 | -0.07 | 0.005 | 5.13E-48 | urate measurement |
| rs10896028 | 11 | 65,432,187 | a | t | 0.645 | -0.048 | 0.004 | 4.10E-33 | urate measurement |
| rs7315236 | 12 | 52,251,933 | t | c | 0.357 | 0.029 | 0.004 | 1.91E-13 | urate measurement |
| rs12313306 | 12 | 57,751,854 | t | c | 0.246 | -0.076 | 0.004 | 6.74E-65 | urate measurement; glomerular filtration rate |
| rs1800574 | 12 | 121,416,864 | t | c | 0.031 | -0.081 | 0.012 | 2.84E-12 | [type ii diabetes mellitus](https://www.ebi.ac.uk/gwas/efotraits/EFO_0001360); [urate measurement](https://www.ebi.ac.uk/gwas/efotraits/EFO_0004531); [creatinine measurement](https://www.ebi.ac.uk/gwas/efotraits/EFO_0004518); [glomerular filtration rate](https://www.ebi.ac.uk/gwas/efotraits/EFO_0005208); [IgF-1 measurement](https://www.ebi.ac.uk/gwas/efotraits/EFO_0004627); [serum gamma-glutamyl transferase measurement](https://www.ebi.ac.uk/gwas/efotraits/EFO_0004532); [sex hormone-binding globulin measurement](https://www.ebi.ac.uk/gwas/efotraits/EFO_0004696); [blood phosphate measurement](https://www.ebi.ac.uk/gwas/efotraits/EFO_0010972) |
| rs28530689 | 12 | 122,500,748 | a | c | 0.512 | 0.032 | 0.004 | 1.27E-16 | [body weight](https://www.ebi.ac.uk/gwas/efotraits/EFO_0004338); [urate measurement](https://www.ebi.ac.uk/gwas/efotraits/EFO_0004531) |
| rs12423664 | 12 | 133,069,894 | a | g | 0.152 | 0.042 | 0.006 | 1.75E-13 | [urate measurement](https://www.ebi.ac.uk/gwas/efotraits/EFO_0004531); [coronary artery disease](https://www.ebi.ac.uk/gwas/efotraits/EFO_0001645), [triglyceride measurement](https://www.ebi.ac.uk/gwas/efotraits/EFO_0004530); Lipid traits; [hypertension](https://www.ebi.ac.uk/gwas/efotraits/EFO_0000537); [diastolic blood pressure](https://www.ebi.ac.uk/gwas/efotraits/EFO_0006336); [systolic blood pressure](https://www.ebi.ac.uk/gwas/efotraits/EFO_0006335) |
| rs7986094 | 13 | 31,029,931 | a | c | 0.302 | -0.024 | 0.004 | 1.74E-08 | [urate measurement](https://www.ebi.ac.uk/gwas/efotraits/EFO_0004531) |
| rs626277 | 13 | 72,347,696 | a | c | 0.594 | 0.026 | 0.004 | 2.69E-11 | [urate measurement](https://www.ebi.ac.uk/gwas/efotraits/EFO_0004531); [creatinine measurement](https://www.ebi.ac.uk/gwas/efotraits/EFO_0004518), [chronic kidney disease](https://www.ebi.ac.uk/gwas/efotraits/EFO_0003884); [uric acid measurement](https://www.ebi.ac.uk/gwas/efotraits/EFO_0004761); [glomerular filtration rate](https://www.ebi.ac.uk/gwas/efotraits/EFO_0005208) |
| rs861536 | 14 | 104,167,564 | a | g | 0.621 | 0.024 | 0.004 | 2.16E-09 | [urate measurement](https://www.ebi.ac.uk/gwas/efotraits/EFO_0004531) |
| rs1478604 | 15 | 39,873,321 | t | c | 0.706 | -0.026 | 0.004 | 4.49E-10 | [urate measurement](https://www.ebi.ac.uk/gwas/efotraits/EFO_0004531) |
| rs2929508 | 15 | 72,246,964 | a | t | 0.261 | -0.029 | 0.005 | 3.65E-09 | [urate measurement](https://www.ebi.ac.uk/gwas/efotraits/EFO_0004531) |
| rs8040109 | 15 | 73,334,225 | a | c | 0.707 | 0.025 | 0.004 | 5.85E-09 | [urate measurement](https://www.ebi.ac.uk/gwas/efotraits/EFO_0004531) |
| rs2472297 | 15 | 75,027,880 | t | c | 0.249 | -0.028 | 0.005 | 1.50E-08 | [glomerular filtration rate](https://www.ebi.ac.uk/gwas/efotraits/EFO_0005208); [urate measurement](https://www.ebi.ac.uk/gwas/efotraits/EFO_0004531); [caffeine metabolite measurement](https://www.ebi.ac.uk/gwas/efotraits/EFO_0007872); [alcohol consumption measurement](https://www.ebi.ac.uk/gwas/efotraits/EFO_0007878); [sodium measurement](https://www.ebi.ac.uk/gwas/efotraits/EFO_0009282); [urinary albumin to creatinine ratio](https://www.ebi.ac.uk/gwas/efotraits/EFO_0007778); [creatinine measurement](https://www.ebi.ac.uk/gwas/efotraits/EFO_0004518); [coffee consumption measurement](https://www.ebi.ac.uk/gwas/efotraits/EFO_0006781); tea consumption measurement; response to clozapine; [potassium measurement](https://www.ebi.ac.uk/gwas/efotraits/EFO_0009283); [albuminuria](https://www.ebi.ac.uk/gwas/efotraits/EFO_0004285), etc. |
| rs57737646 | 15 | 76,299,828 | t | c | 0.025 | -0.094 | 0.012 | 5.40E-14 | [urate measurement](https://www.ebi.ac.uk/gwas/efotraits/EFO_0004531) |
| rs55781567 | 15 | 78,857,986 | c | g | 0.655 | 0.023 | 0.004 | 1.11E-08 | [lung carcinoma](https://www.ebi.ac.uk/gwas/efotraits/EFO_0001071): [forced expiratory volume](https://www.ebi.ac.uk/gwas/efotraits/EFO_0004314), [response to bronchodilator](https://www.ebi.ac.uk/gwas/efotraits/GO_0097366); [non-small cell lung carcinoma](https://www.ebi.ac.uk/gwas/efotraits/EFO_0003060); [FEV/FEC ratio](https://www.ebi.ac.uk/gwas/efotraits/EFO_0004713), [response to bronchodilator](https://www.ebi.ac.uk/gwas/efotraits/GO_0097366): [lung adenocarcinoma](https://www.ebi.ac.uk/gwas/efotraits/EFO_0000571): [skin aging measurement](https://www.ebi.ac.uk/gwas/efotraits/EFO_0008006): [urate measurement](https://www.ebi.ac.uk/gwas/efotraits/EFO_0004531); [squamous cell lung carcinoma](https://www.ebi.ac.uk/gwas/efotraits/EFO_0000708): [upper aerodigestive tract neoplasm](https://www.ebi.ac.uk/gwas/efotraits/EFO_0004284) |
| rs4997081 | 16 | 20,365,234 | c | g | 0.196 | -0.03 | 0.005 | 4.18E-10 | [urate measurement](https://www.ebi.ac.uk/gwas/efotraits/EFO_0004531) |
| rs8050136 | 16 | 53,816,275 | a | c | 0.403 | 0.025 | 0.004 | 2.34E-10 | [body mass index](https://www.ebi.ac.uk/gwas/efotraits/EFO_0004340); [type ii diabetes mellitus](https://www.ebi.ac.uk/gwas/efotraits/EFO_0001360); [physical activity measurement](https://www.ebi.ac.uk/gwas/efotraits/EFO_0008002); [age at menarche](https://www.ebi.ac.uk/gwas/efotraits/EFO_0004703); [blood urea nitrogen measurement](https://www.ebi.ac.uk/gwas/efotraits/EFO_0004741); [urate measurement](https://www.ebi.ac.uk/gwas/efotraits/EFO_0004531); [subcutaneous adipose tissue measurement](https://www.ebi.ac.uk/gwas/efotraits/EFO_0004766) |
| rs4788815 | 16 | 71,634,811 | a | t | 0.357 | -0.026 | 0.004 | 7.44E-11 | [urate measurement](https://www.ebi.ac.uk/gwas/efotraits/EFO_0004531); [metabolite measurement](https://www.ebi.ac.uk/gwas/efotraits/EFO_0004725); [type ii diabetes mellitus](https://www.ebi.ac.uk/gwas/efotraits/EFO_0001360); [coronary artery calcification](https://www.ebi.ac.uk/gwas/efotraits/EFO_0004723) |
| rs57652769 | 16 | 79,753,976 | t | c | 0.309 | -0.036 | 0.004 | 8.56E-18 | [urate measurement](https://www.ebi.ac.uk/gwas/efotraits/EFO_0004531); [platelet count](https://www.ebi.ac.uk/gwas/efotraits/EFO_0004309); [aspartate aminotransferase measurement](https://www.ebi.ac.uk/gwas/efotraits/EFO_0004736); [serum alanine aminotransferase measurement](https://www.ebi.ac.uk/gwas/efotraits/EFO_0004735); [goiter](https://www.ebi.ac.uk/gwas/efotraits/EFO_0004283) |
| rs9925837 | 16 | 79,927,303 | a | g | 0.845 | -0.042 | 0.005 | 5.85E-15 | [urate measurement](https://www.ebi.ac.uk/gwas/efotraits/EFO_0004531); [fibroblast growth factor 23 measurement](https://www.ebi.ac.uk/gwas/efotraits/EFO_0009381) |
| rs11644696 | 16 | 81,572,093 | a | g | 0.477 | 0.022 | 0.004 | 1.41E-08 | [urate measurement](https://www.ebi.ac.uk/gwas/efotraits/EFO_0004531) |
| rs2453580 | 17 | 19,438,321 | t | c | 0.598 | 0.025 | 0.004 | 7.01E-10 | [urate measurement](https://www.ebi.ac.uk/gwas/efotraits/EFO_0004531); [glomerular filtration rate](https://www.ebi.ac.uk/gwas/efotraits/EFO_0005208), [creatinine measurement](https://www.ebi.ac.uk/gwas/efotraits/EFO_0004518) |
| rs57070985 | 19 | 4,969,053 | a | g | 0.646 | 0.029 | 0.004 | 2.04E-12 | [urate measurement](https://www.ebi.ac.uk/gwas/efotraits/EFO_0004531) |
| rs4808762 | 19 | 18,326,222 | t | c | 0.72 | -0.024 | 0.004 | 1.36E-08 | [urate measurement](https://www.ebi.ac.uk/gwas/efotraits/EFO_0004531); [visceral adipose tissue measurement](https://www.ebi.ac.uk/gwas/efotraits/EFO_0004765); [aspartate aminotransferase to alanine aminotransferase ratio](https://www.ebi.ac.uk/gwas/efotraits/EFO_0010934); [total cholesterol measurement](https://www.ebi.ac.uk/gwas/efotraits/EFO_0004574); [serum gamma-glutamyl transferase measurement](https://www.ebi.ac.uk/gwas/efotraits/EFO_0004532) |
| rs2868194 | 19 | 33,350,060 | t | c | 0.408 | -0.027 | 0.004 | 8.90E-12 | [urate measurement](https://www.ebi.ac.uk/gwas/efotraits/EFO_0004531) |
| rs10414501 | 19 | 50,259,674 | c | g | 0.957 | -0.125 | 0.018 | 1.56E-12 | [urate measurement](https://www.ebi.ac.uk/gwas/efotraits/EFO_0004531) |
| rs7267595 | 20 | 10,643,850 | a | c | 0.51 | 0.023 | 0.004 | 3.15E-09 | [urate measurement](https://www.ebi.ac.uk/gwas/efotraits/EFO_0004531); [heel bone mineral density](https://www.ebi.ac.uk/gwas/efotraits/EFO_0009270) |
| rs6119510 | 20 | 33,287,782 | t | g | 0.596 | -0.023 | 0.004 | 3.20E-09 | [urate measurement](https://www.ebi.ac.uk/gwas/efotraits/EFO_0004531) |
| rs142773928 | 20 | 43,038,720 | a | g | 0.166 | 0.032 | 0.005 | 3.77E-09 | [urate measurement](https://www.ebi.ac.uk/gwas/efotraits/EFO_0004531) |
| rs1800961 | 20 | 43,042,364 | t | c | 0.034 | -0.076 | 0.012 | 1.63E-10 | [high density lipoprotein cholesterol measurement](https://www.ebi.ac.uk/gwas/efotraits/EFO_0004612); [c-reactive protein measurement](https://www.ebi.ac.uk/gwas/efotraits/EFO_0004458); [total cholesterol measurement](https://www.ebi.ac.uk/gwas/efotraits/EFO_0004574); [type ii diabetes mellitus](https://www.ebi.ac.uk/gwas/efotraits/EFO_0001360); [neutrophil count](https://www.ebi.ac.uk/gwas/efotraits/EFO_0004833); [low density lipoprotein cholesterol measurement](https://www.ebi.ac.uk/gwas/efotraits/EFO_0004611); [hemoglobin measurement](https://www.ebi.ac.uk/gwas/efotraits/EFO_0004509); [hematocrit](https://www.ebi.ac.uk/gwas/efotraits/EFO_0004348); [alcohol drinking](https://www.ebi.ac.uk/gwas/efotraits/EFO_0004329); [urate measurement](https://www.ebi.ac.uk/gwas/efotraits/EFO_0004531), etc. |
| rs219781 | 21 | 37,832,621 | t | g | 0.246 | -0.025 | 0.004 | 1.56E-08 | [urate measurement](https://www.ebi.ac.uk/gwas/efotraits/EFO_0004531); [calcium measurement](https://www.ebi.ac.uk/gwas/efotraits/EFO_0004838) |
| rs12485100 | 22 | 44,325,516 | t | g | 0.173 | -0.033 | 0.005 | 2.44E-10 | [urate measurement](https://www.ebi.ac.uk/gwas/efotraits/EFO_0004531) |

SNP, single nucleotide polymorphisms; GWAS, genome-wide association study; Effect: effect of the SNP; SE: standard error

| Supplementary Table3 Summary of index SNPs identified in previous gout GWAS used as genetic instruments for gout | | | | | | | | | |
| --- | --- | --- | --- | --- | --- | --- | --- | --- | --- |
| SNP | Chromo-some | Position (b37) | Coded allele | Non-coded allele | Coded allele freq | Effect | SE | P-value | Reported traits in GWAS Catalog |
| rs79945326 | 2 | 27170093 | a | c | 0.0595 | 0.158 | 0.0284 | 2.55E-08 | gout |
| rs141982039 | 4 | 9704769 | t | c | 0.974 | 0.2675 | 0.0467 | 1.01E-08 | gout |
| rs75674432 | 4 | 10189768 | t | c | 0.9804 | -0.3072 | 0.0539 | 1.19E-08 | gout |
| rs1229984 | 4 | 100239319 | t | c | 0.0345 | 0.3822 | 0.04 | 1.27E-21 | gout, alcohol consumption measurement; esophageal carcinoma; body mass index; low density lipoprotein cholesterol measurement; vitamin D measurement; blood urea nitrogen measurement, etc. |
| rs28660692 | 11 | 26687055 | a | c | 0.5973 | 0.0752 | 0.0131 | 1.04E-08 | gout |
| rs112770252 | 18 | 30119124 | a | g | 0.987 | -0.342 | 0.0622 | 3.89E-08 | gout |

SNP, single nucleotide polymorphisms; GWAS, genome-wide association study; Effect: effect of the SNP; SE: standard error.

| Supplementary Table 4 Incidence rate and Hazard ratios of any cardiovascular disease (CVD) among patients with studied hyperuricemia or gout when compared with patients without hyperuricemia and gout, studied among White participants | | | | | |
| --- | --- | --- | --- | --- | --- |
|  | Hyperuricemia | | | Gout | |
| Any CVD | Incidence Rate | HR (95% CI) | Incidence Rate | | HR (95% CI) |
| Unexposed group | 25,852/3,117.77 (8.29) | Ref | 2,5852/3,117.77 (8.29) | | Ref |
| Exposed group | 6,965/509 (13.68) | 1.32 (1.28-1.35) | 1,731/89.08 (19.43) | | 1.51 (1.44-1.59) |

| Supplementary Table 5 Association of hyperuricemia and gout with risk of CVD^1^ using Mendelian Randomization (MR) analyses, after excluding SNPs associated with other traits (with therefore possible horizontal pleiotropy) | | | | | | |
| --- | --- | --- | --- | --- | --- | --- |
|  | One-sample MR analysis | | Two-sample MR analysis | | |  |
| Exposure | OR (95%CI)^4^ | P | | OR (95%CI)^4^ | P | |
| Hyperuricemia^2^ | 1.11 (1.04-1.19) | 0.005 | | 1.17 (1.04-1.27 | < 0.001 | |
| Gout^3^ | 0.98 (0.79-1.12) | 0.806 | | 1.00 (0.90-1.10) | 0.949 | |

^1^A primary diagnosis of CVD in UK Biobank inpatient data, or a death with CVD as the underlying cause, according to UK Biobank mortality data (ICD-10: I00-I70, I730, and I74).

^2^Hyperuricemia: 44 independent SNPs derived from published GWAS data (not related to gout or any other traits) were used as instrumental variables to infer causality.

^3^Gout: 5 independent SNPs were used instrumental variables, which was not related to hyperuricemia or any other traits.

^4^OR, Odds ratio; CI, confidence interval; models adjusted for sex, age, genotyping array, and 5 PCs.


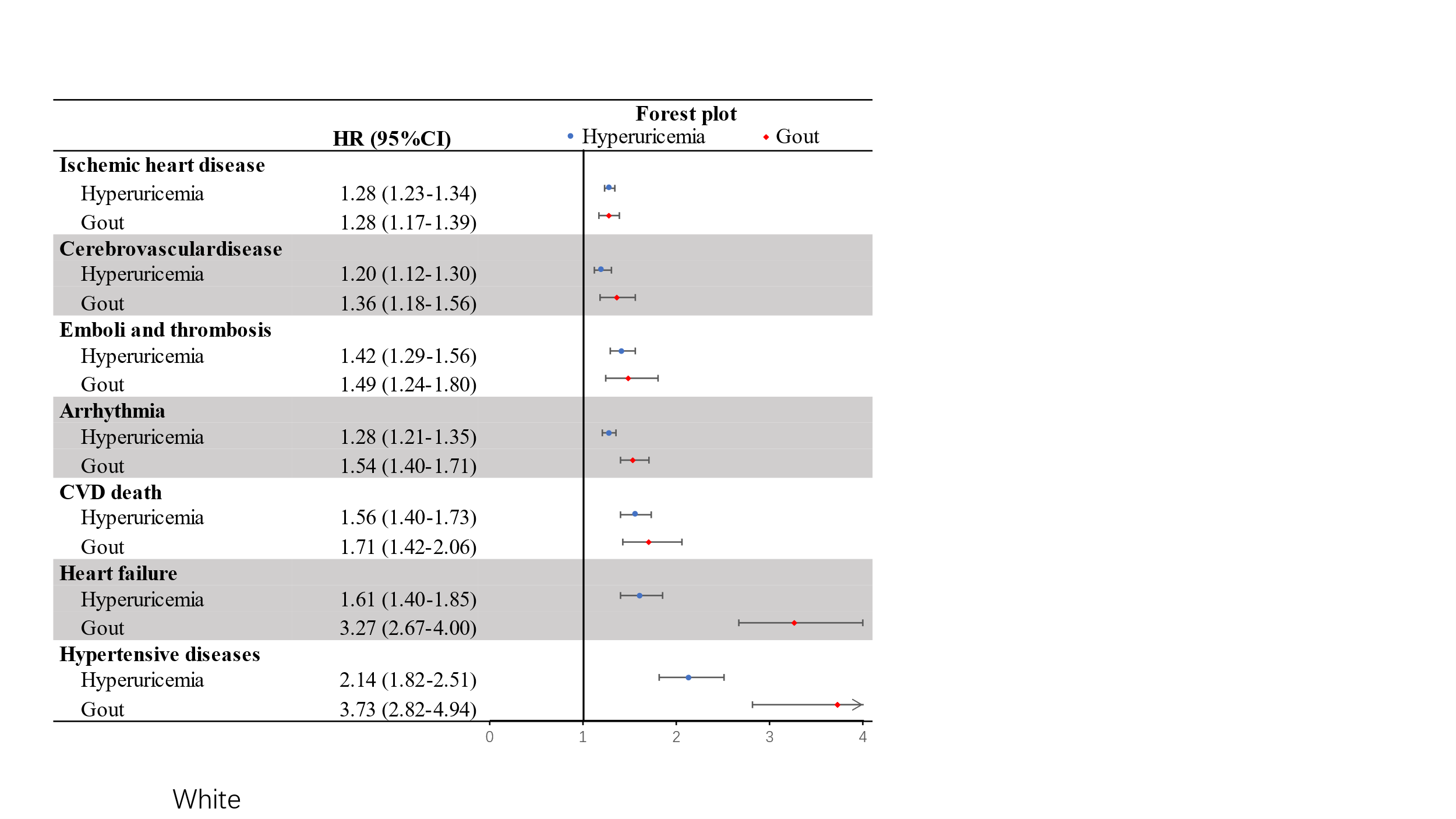
Supplementary Figure 1 Hazard ratios of any or specific cardiovascular diseases (CVDs) among patients with studied hyperuricemia or gout when compared with patients without hyperuricemia and gout, studied among White participants.


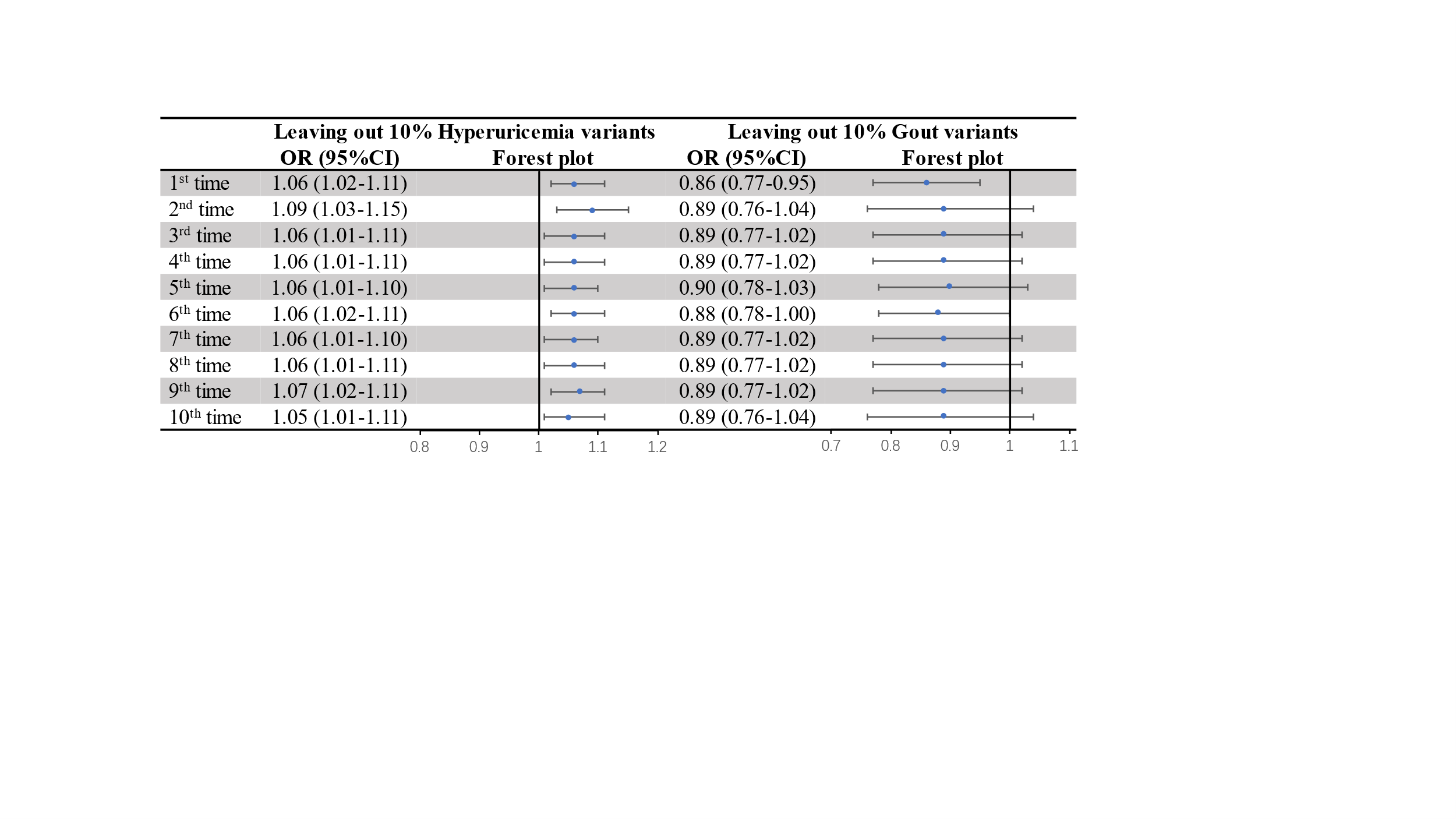
Supplementary Figure 2 Analysis of effect of hyperuricemia, gout on cardiovascular diseases using IVW method, after leaving out a randomly chosen 10% variants
